# Epigenetic Aspects of Systemic Lupus Erythematosus

**DOI:** 10.1007/s40744-015-0014-y

**Published:** 2015-06-16

**Authors:** Manfred Relle, Bernd Foehr, Andreas Schwarting

**Affiliations:** Department of Medicine I, Mainz University Medical Center, Langenbeckstrasse 1, 55131 Mainz, Germany

**Keywords:** Epigenetics, Gene imprinting, Histone modifications, Methylation, Non-coding RNA, Nucleosome remodeling, Systemic lupus erythematosus (SLE)

## Abstract

Autoimmune diseases such as systemic lupus erythematosus (SLE), rheumatoid arthritis, multiple sclerosis, autoimmune hepatitis, and inflammatory bowel disease have complex pathogeneses and the courses of events leading to these diseases are not well understood. The immune surveillance is a delicate balance between self and foreign as well as between tolerance and immune response. Exposure to certain environmental factors may impair this equilibrium, leading to autoimmune diseases, cancer, and the so-called “lifestyle diseases” such as atherosclerosis, heart attack, stroke, and obesity, among others. These external stimuli may also alter the epigenetic status quo and may trigger autoimmune diseases such as SLE in genetically susceptible individuals. This review aims to highlight the role of epigenetic (dys-)regulation in the pathogenesis of SLE.

**Electronic supplementary material:**

The online version of this article (doi:10.1007/s40744-015-0014-y) contains supplementary material, which is available to authorized users.

## Introduction

One interesting hypothesis concerning autoimmune diseases is that environmental effects on immune responses could be mediated by alterations in the epigenetic profile. Indeed, there is evidence that environmental factors may be the reason for the high discordance rate for autoimmune diseases in identical twins [1–4]. Advances in molecular genetics have illustrated that genomes are not a static entity for the deposition of genetic information. These findings imply dynamic response to external stimuli and a high genomic plasticity that is affected by epigenetic gene regulation. This kind of gene regulation relies on inducible and/or heritable patterns of gene expression, which are not based on changes of the genomic DNA sequence. Major mechanisms include DNA methylation, histone modification, non-coding RNA expression, gene imprinting and chromatin remodeling. Although in neither of these cases is gene expression modified by changes in the base sequence, these mechanisms interact with each other in a complex manner to regulate the expression and silencing of genes.

As epigenetic gene regulation is a new and highly promising research field (in autoimmune diseases), this article reviews the role of epigenetic regulation in systemic lupus erythematosus (SLE). This article is based on previously conducted studies and does not involve any new studies of human or animal subjects performed by any of the authors.

## What is Epigenetics?

Although known before, the word “epigenetics” was introduced in modern science in 1942 by Conrad Hal Waddington, a British developmental biologist [5]. The concept of epigenetics is defined as the study of regulatory mechanisms that account for (potentially) heritable and reversible patterns in gene expression without affecting the nucleotide sequence of the genome. As the Greek prefix “ “ (epi) means “upon, over, on top of”, the “epigenome” is thought to be an additional, secondary informational level on top of the genetic code. A classic example of epigenetic regulation in mammals is the dosage compensation by silencing of one X chromosome in females. A condensed chromatin configuration prevents expression of genes on the silenced X chromosome, while the other X chromosome in the same nucleus is actively transcribed [6, 7]. It has been shown in humans that the silencing of individual gene loci by imprinting (in combination with micro deletions and mutations) leads to developmental abnormalities, known as Beckwith–Wiedemann, Angelman and Prader–Willi syndromes [8–10].

As the molecular basis of inheritance was unknown at that time, the term was initially used in an unspecific sense. This in conjunction with the description of the DNA double-helix structure by Watson and Crick, which demonstrated its eminent role in inheritance [11], “have cast a shadow over this discipline for decades” [12]. The term was reintroduced no more than four decades later, as studies on chromatin structure had identified the molecular basis of epigenetics.

The year 1974 marked the “birth-year” of modern, molecular-based epigenetics. There, Kornberg and colleagues published that chromatin is “a repeating unit of histones and DNA” [13]. These repetitive units were then called “nucleosomes”. However, it took until 1996 before two studies provided the first clear connection between histone acetylation and transcriptional regulation [14, 15] and it took further 4 years before a functional link between histone methylation and chromatin structure could be established [16].

In transcription and DNA replication, changes in the chromatin structure are essential to overcome steric hindrances for DNA binding factors [17–19]. These fundamental alterations are called “chromatin remodeling”. Chromatin remodeling is, amongst other things, based on post-translational modifications of histones by histone-modifying enzymes, which induce a complex cascade of post-translational modifications that can either activate or repress transcription [20]. This has led to the notion that defined patterns of histone modifications alter the structure of higher order chromatin to recruit effector molecules [21]. The different combinations of these modifications are thought to constitute a code, which has led to the so-called “histone code” hypothesis [20].

## General Epigenetic Mechanisms

Among the approximately 3 billion base pairs of the mammalian genome, there are 20,000–30,000 protein-coding genes [22–24], which need instructions for where and when to be expressed or silenced. Only the accurate interplay of these genes results in functioning cells, organs and organisms. Like cancer and other complex diseases, autoimmune diseases seem to be the result of multistep processes in which genetic predisposition and epigenetic alterations interact and contribute to the pathological changes of this susceptible interplay.

**Fig. 1:**
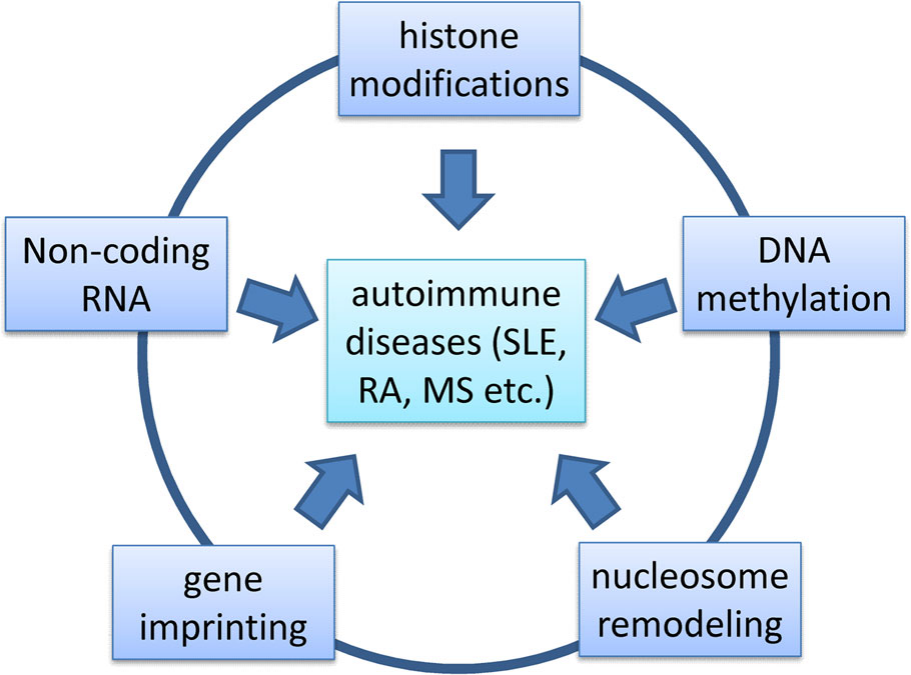
Epigenetics and autoimmune disease. Five different epigenetic mechanisms have been identified so far: histone modification, non-coding RNA expression, DNA methylation, gene imprinting, and chromatin remodeling. These mechanisms interact with each other in a complex manner to regulate the expression and silencing of genes. Environmental factors may be the reason for the (high) discordance rate for autoimmune diseases in identical twins. These environmental factors may alter the epigenetic status quo and may trigger autoimmune diseases, including systemic lupus erythematosus (SLE), rheumatoid arthritis (RA), multiple sclerosis (MS), inflammatory bowel disease, as well as autoimmune diabetes, thyroid disease and hepatitis

Two major groups of cellular compounds are affected by epigenetic changes: the genome and the histones. Major mechanisms of epigenetic gene regulation include DNA methylation, histone modification, non-coding RNA expression, gene imprinting, and chromatin remodeling (Fig. 1). Mainly, three types of epigenetic modifications of chromatin transition are known: histone hypoacetylation, methylation of lysine 9 of histone H3 [Me(Lys9)H3], and DNA methylation at CpG dinucleotides. However, Me(Lys9)H3 is typically not found on regulated genes. It is characteristic for so-called constitutive heterochromatin, whereas genes that are shut off are typically localized in ‘facultative’ heterochromatin, which is marked by tri-methylated lysine 27 on histone H3 (H3K27me3).

Histones are subject to diverse post-translational modifications including acetylation, methylation, phosphorylation, and ubiquitylation [21, 25, 26]. In 1996, Taunton et al. [15] identified the first histone deacetylase (HDAC), a human homolog of the yeast transcriptional regulator Rpd3p. Since that time, large and ancient families of HDACs have been identified in yeast as well as in mammals [27–29]. It is believed that HDACs reverse the regulatory acetylation of histone proteins and silence genes by stabilizing a transcription-incompetent condition of nucleosomes [27]. It has been demonstrated in animal models that HDAC inhibitors are therapeutic for several inflammatory diseases [30], suggesting a potential therapeutic use of these inhibitors in autoimmune diseases.

Methylation of lysine 9 of histone H3 is catalyzed by the mammalian homolog of the fruit fly suppressor of variegation 3–9 [Su(var)3–9], which is a histone lysine methyltransferase that selectively methylates histone H3 at this site [16]. This modification generates a binding site for HP1 proteins, a family of heterochromatic transcriptional repressors that establish a repressed chromatin state [31–33], which is also called “gene silencing”.

DNA methylation patterns are established and maintained by methyltransferases [34]. Any deletion of these enzymes is lethal during embryogenesis [35–37]. Methylated cytosines within CpG dinucleotides are recognized by methyl-CpG binding proteins that recruit HDACs and thereby induce an inhibitory chromatin configuration [38]. Histone and DNA modifications are highly dynamic, a property that is crucial for the regulation and control of cellular proliferation, differentiation and survival [39]. Moreover, they are substantial constituents of the so-called “epigenetic code” [40].

In the last three decades, major advances have been made in understanding the interaction between DNA methylation, histone modification, and gene expression. This fundamental research demonstrated that the interplay between the individual components is highly complex and opened the new field of epigenetics. In the last few years, it became evident that epigenetic changes are not only involved in cancer and developmental processes but also play a significant role in the etiopathology of autoimmune diseases.

## Epigenetic Changes in Autoimmune Diseases

**Fig. 2:**
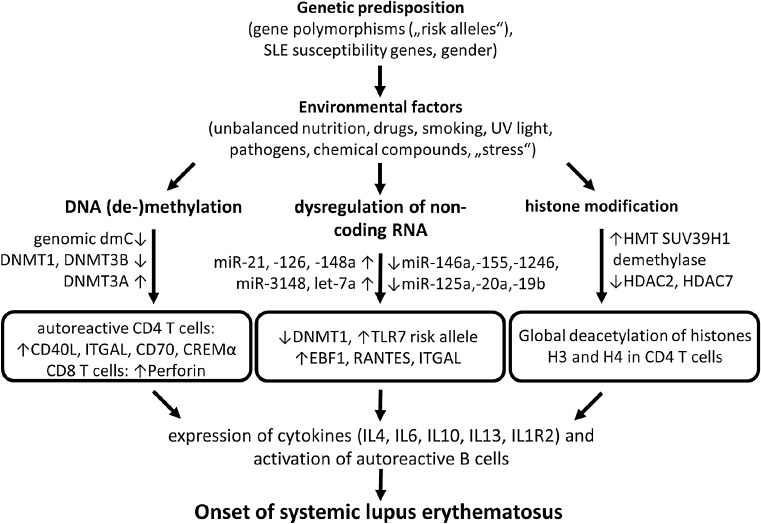
Possible roles of epigenetic alterations involved in SLE pathogenesis. Genetic disposition in combination with environmental factors can alter epigenetic marks, such as DNA (de-)methylation, histone modifications and transcriptional regulation by non-coding RNA. These epigenetic modifications may lead to aberrant gene expression profiles in (autoreactive) T cells that activate proinflammatory and repress anti-inflammatory genes. Aberrant and continuous expression of chemokines and cytokines mobilizes autoreactive B cells, which may trigger and aggravate SLE. *SLE* systemic lupus erythematosus

The mechanisms underlying epigenetic changes are of great importance to human autoimmune diseases. However, they are poorly understood (Fig. 2). Over the years, increasing evidence has demonstrated the important role for aberrant epigenetics in the pathogenesis of SLE [41].

A number of genes have been claimed to be associated with susceptibility to anti-self responses. Because of their considerable heterogeneity, the immunoglobulin genes, the T cell receptor genes and the major histocompatibility complex (MHC) genes have soon been suspected of playing a distinct role in the pathology of SLE and other autoimmune diseases. In the past few years, progress has been made in identifying SLE susceptibility genes in mice [42]. However, these models only try to decipher the complex genetic background of SLE, but not the comparably complex environmental background of the disease. In general, mice are housed under (standardized) specific pathogen-free conditions and, thus, do not fully resemble the human disease. Interestingly, the data on several autoimmune diseases (including SLE) disclose increasing disease rates over the past few decades [43]. As genetic determinants are unlikely to alter disease rates within such short intervals, any rapid change indicates an environmental influence [43].

Since Holliday and Pugh proposed that “cytoplasmic components can have a powerful or overriding influence on genomic activity” in 1975 [44], many investigations broadened our understanding of epigenetic alterations in the pathogenesis of various complex disorders, including cancer and autoimmunity. One important epigenetic mechanism is the cytosine methylation/demethylation “switch” of regulatory DNA sequences. Highly simplified, methylation inactivates transcription, while hypomethylation associated with the activation of genes [45–47]. CpG dinucleotides are found at a lower frequency in the genome than would be expected due to random distribution, a phenomenon called “CG suppression”. This loss of CpGs has been explained by a spontaneous deamination of methylated cytosine residues [48]. In contrast, regions with a higher CpG content are found in approximately 40% of mammalian promoters [49]. These accumulations are called “CpG islands” [38, 50] and in most instances the CpG sites of these CpG islands are unmethylated if the genes are expressed. Environmentally induced hypomethylation of proinflammatory genes on the one hand and hypermethylation of anti-inflammatory genes on the other hand may have the effect that genetically susceptible individuals come down with an autoimmune disease.

## How is SLE Influenced by Epigenetic Changes?

T cells from patients with active SLE have a 17% decrease in genomic deoxymethylcytosine content [51] and the inhibition of T cell DNA methylation causes autoreactivity in vitro and a SLE-like disease in vivo [51]. In murine models of drug-induced lupus erythematosus, it has been shown that mice receiving CD4+ T cells treated with demethylating agents, including procainamide and hydralazine, develop a SLE-like disease [52]. These drugs also inhibit T cell DNA methylation and induce autoreactivity in cloned T cell lines [53, 54]. CD4+ T cells treated with DNA methylation inhibitor 5-azacytidine become autoreactive and respond to self-class II MHC without the addition of exogenous antigen [55]. In lupus-prone MRL/lpr mice, defective DNA methylation and CD70 overexpression in CD4+ T cells could be detected [56].

It is well known that an exposure to ultraviolet light can trigger lupus flares [57, 58]. Moreover, brief exposures of T cells to ultraviolet light induce DNA hypomethylation and T cell autoreactivity [59], which supports an association between T cell DNA hypomethylation and autoimmunity [60]. Lymphocyte function-associated antigen-1 (LFA1) is a heterodimer consisting of the integrin alpha L (ITGAL) and the beta 2 chain (ITGB2), which is expressed on all leukocytes. In T cells from SLE, patients’ sequences flanking the ITGAL gene promoter region were demethylated, suggesting a mechanism for LFA-1 overexpression on an autoreactive subset of T cells [51]. Indeed, overexpression of LFA-1 [60, 61] and CD70 (TNFSF7) [62, 63], which, in turn, induces autoantibody synthesis in B cells [64], is thought to be involved in T cell autoreactivity in SLE. Additional studies have confirmed that DNA hypomethylation and histone hyperacetylation of CD11a and CD70 promoter regions contribute to their overexpression in SLE CD4+ T cells [51, 65, 66]. Hypermethylation of MHC class II transactivator (MHC2TA) and downregulation of human leukocyte antigen (HLA)-DR and MHC2TA could also be observed [67]. Different blood cell populations of SLE patients are characterized by a global loss of DNA methylation [68]. For instance, persistent hypomethylation of interferon genes and interferon-regulated genes can be found in CD4+ T cells [69], CD19+ B cells, CD14+ monocytes [70], and neutrophils [71] of patients with SLE. This process is associated with defects in extracellular-signal-regulated kinases (ERK) pathway signaling and consequent downregulation of the methyltransferase DNMT 1 [72]. These studies indicate that T cells hypomethylated by treatment with DNA methyltransferase inhibitors or ERK pathway inhibitors are sufficient to induce a SLE-like disease [73]. Recently, it could be shown that female but not male mice with an inducible ERK defect developed SLE-like symptoms in a transgenic mouse model, demonstrating ERK-dependent female predisposition for SLE [74]. Gorelik et al. [75] traced the SLE ERK pathway defect to impaired protein kinase C delta (PKCδ) phosphorylation. Additionally identified demethylation targets in SLE are genes involved in inflammation (*CD40LG*) [74, 76], cytokine pathway (IL-4 [77, 78], IL-6 [77, 79], IL-10 [80], IL-13 [80]) and IL1R2 [81], respectively) and cell lysis (perforin [82, 83]), which all can increase inflammation by stimulating the immune system.

Another example concerns the overexpression of the transcription regulatory factor cAMP-responsive element modulator alpha (CREMα) in T cells of patients with SLE and lupus-prone MRL/lpr mice. It binds to the CRE site in the promoter region of genes and contributes to epigenetic remodeling through the recruitment of DNA methyltransferase DNMT3A [84]. Then, DNMT3A mediates CpG hypomethylation, remodeling the CD8 cluster [85] and silencing of IL2 and IL17A [84]. On the other hand, it has recently been shown, that an increased histone H3 lysine 27 trimethylation enrichment at the hematopoietic progenitor kinase 1 (HPK1) promoter of SLE CD4+ T cells (relative to controls) inhibits the HPK1 expression and contributes to autoimmunity in SLE [86]. All these reports support a role for epigenetic DNA alterations in the pathogenesis of SLE.

However, DNA methylation is not a process whose effects are restricted to the DNA. As the methylation of DNA maintains chromatin in a condensed and hence, more inactive configuration, it acts synergistically or antagonistically on the diverse modifications of histone proteins [87]. For instance, hypomethylated CpG island chromatin is enriched in hyperacetylated histones and deficient in linker histones [88]. DNA methylation may also protect individuals from autoimmune diseases, such as SLE: as the estrogen receptor becomes hypermethylated during aging [89], this change may reduce the risk for women to come down with SLE or other sex-related autoimmune diseases. The identification of further genes that are deregulated by DNA (de-)methylation will successively account for a better understanding of the aberrant physiological pathways of SLE.

CD4+ lymphocytes undergo global histone H3 and H4 deacetylation and consequent skewed gene expression [68]. However, currently little is known about the relationship between alterations of histone modification patterns and SLE. However, histone H3 and H4 hypoacetylation as well as a lysine 9 on H3 (H3K9) hypomethylation have been described for SLE-derived CD4+ cells [90]. The same group observed increased histone H3 acetylation and dimethylated H3 lysine 4 (H3K4me2) levels in the *TNFSF7* (CD70) gene promoter region in SLE CD4+ T cells, which correlated positively with disease activity [91]. Histone acetylation is regulated by histone acetyltransferases (HATs) and HDACs [92, 93]. Previous studies have shown that HDAC2 and HDAC7 levels are downregulated in active lupus erythematosus CD4+ T cells [90] and HDAC7 is decreased in MRL-lpr/lpr mice [94]. Moreover, it could be shown that the specific class I and II HDAC inhibitor ITF2357 was able to ameliorate SLE-like symptoms in NZB/W mice through regulation of T cell profiles [95].

Further evidence for a substantial role of histone modification in SLE pathogenesis comes from Lu and coworkers. They found that the transcription factor regulatory factor X-box 1 (RFX1) regulates CD70 and CD11a expression in T cells of patients with SLE by recruiting histone methyltransferase SUV39H1 [96]. The downregulation of RFX1 contributes to DNA hypomethylation and histone H3 hyperacetylation at the CD11a and CD70 promoters in CD4+ T cells of patients with SLE, which trigger immune responses [66].

A recent study detected a correlation between the 5-hydroxymethylcytosine (5-hmC) level in the peripheral blood and SLE [97]. The oxidation of 5-methylcytosine (5-mC) to 5-hmC is an epigenetic mechanism which is present in the DNA of mammalian cells. First seen in bacteriophages in 1952 [98], it was then found in high levels in neurons of the central nervous system in human and mouse as well as in embryonic stem cells [99, 100]. The exact function of this sixth DNA base is not fully understood, but it is thought to regulate gene expression and prompt DNA demethylation. This hypothesis is supported by the observation that hydroxylation of 5-mC to 5-hmC by TET1 actively promotes DNA demethylation [101]. Reduction of hmC levels in DNA is also a hallmark of cancers and, contrary to DNA methylation, which occurs immediately during replication, hmC forms slowly during the first 30 h following DNA synthesis [102].

## Role of Micro RNA in SLE Pathogenesis

Micro RNA (miRNA) was initially discovered in 1993 [103] but little attention was given to these small RNAs until 2001 [104–108]. miRNAs are an important class of endogenous regulatory small RNAs [109, 110] which (amongst others) regulate the expression of genes involved in immune activation [111]. For cancer, it has been demonstrated that the miR-29 family induces DNA hypomethylation by directly targeting DNA methyltransferases thereby leading to a re-expression of hypermethylated silenced tumor suppressor genes [112, 113]. These studies have shown that miRNAs are involved in disease pathogenesis by targeting DNA methylation. miRNAs are also implicated in the pathology of SLE [114, 115]. One of these miRNAs, miR-146a, is a negative regulator of the IFN pathway. Underexpression of miR-146a contributes to alterations in the type I IFN pathway in lupus patients by targeting the key signaling proteins [114]. Dai et al. [116] identified several miRNAs that are differentially expressed in the peripheral blood mononuclear cells of SLE patients whose expression profiling may provide a useful clue for the etiology of SLE. Expression of miR-21 and miR-148a is highly upregulated in CD4+ T cells from both patients with SLE and MRL/lpr mice [117]. These two miRNAs promote CD4+ T cell hypomethylation by repressing DNA (cytosine-5)-methyltransferase 1 (DNMT1) expression and inducing the expression of autoimmune-associated methylation-sensitive genes [117]. Another miRNA that is upregulated in CD4+ T cells from SLE patients is miR-126 [118]. The overexpression of miR-126 in CD4+ T cells from healthy donors caused demethylation and upregulation of the genes ITGAL (encoding CD11a) and CD70, thereby causing T cell and B cell hyperactivity. It could also be shown that the expression of the mir-126 host gene EGFL7 was upregulated in CD4+ T cells from patients with SLE and that the degree of overexpression is associated with the hypomethylation of its promoter [119]. CCL5 (RANTES) is a chemokine expressed by circulating T cells which recruits leukocytes to sites of inflammation. It could be shown that serum levels of RANTES were significantly elevated in patients with SLE when compared with normal controls [120]. miR-125a negatively regulates RANTES expression by targeting KLF13 in activated T cells [121]. Thus, underexpression of miR-125a contributes to the elevated expression of RANTES in SLE. Tissue factor (TF) is the main initiator of the blood coagulation cascade and it could be shown that monocytes of patients with SLE are characterized by a high TF expression and low miR-19b and -20a levels [122]. Reporter assays demonstrated that miR-20a binds to TF mRNA [123]. Thus, downregulation of miR-19b and miR-20a could contribute to increased TF expression provoking the hypercoagulable state characteristic of patients with SLE. Further miRNAs involved in SLE are miRNA-3148 [124], miRNA-1246 [125], and miRNA-let7A [126], respectively. Excessive activation of the innate immune system involving toll-like receptor 7 (TLR7) has been recognized as an important pathogenic mechanism in SLE [127] and miR-3148, with a predicted binding site at the 3′-untranslated region (3′-UTR) of TLR7 mRNA, modulates the allelic expression of this gene. Individuals carrying the G allele of the single nucleotide polymorphism (SNP) rs3853839 in the 3′-UTR of the TLR7 gene exhibited increased TLR7 expression at both the mRNA and protein level and decreased transcript degradation [124]. In contrast, in people bearing the non-risk C allele of this SNP, miR-3148 perfectly matches the 3′-UTR of the TLR7-mRNA, leading to a faster and more effective degradation of non-risk allele containing TLR7 transcripts. For miR-1246, it was shown that its expression was significantly decreased in B cells from SLE patients and that it specifically targeted the 3′-UTR of the early B cell factor 1 (EBF1) mRNA [125]. These findings provide a causal role of miR-1246 in the pathogenesis of SLE: EBF1 contributes to the development, activation, and proliferation of B cells through activation of the AKT signaling pathway. A downregulation of the miR-1246 expression may decrease the degradation rate of the EBF1 mRNA, leading to a B cell overactivation in patients with SLE. Let-7a is implicated in SLE pathogenesis due to its responsiveness to immune stimulation and its reported inflammatory targets [128, 129]. It could be shown that its overexpression may contribute to hyperplasia and a proinflammatory response, including inflammatory mediator production. Recent studies have shown that a significant fraction of miRNAs themselves is regulated by epigenetic mechanisms [130–132], demonstrating the entire complexity of eukaryotic gene regulation.

We now know that changes in DNA methylation, mRNA, and miRNA expression are characteristic for SLE and correlate with the phenotype of this severe disease [133]. However, more studies are required to consolidate the role of miRNAs in SLE pathology. Until then, the question of if these non-coding RNAs are “hope or hype” [134] remains unanswered for SLE.

## Conclusion

Deciphering the contribution of epigenetic alterations to the pathogenesis of SLE will provide promising insights in this complex autoimmune disease. Epigenetic alterations are (potentially) reversible and hence candidates for the development of new therapeutics. However, to attain this goal, many questions remain to be answered in the promising field of epigenetics.

## Electronic supplementary material

Below is the link to the electronic supplementary material.

Supplementary material 1 (PDF 189 kb)
