# Supplementary material for: Epigenetic Aspects of Systemic Lupus Erythematosus
Source: Rheumatol Ther. 2015 Jun 16;2(1):33–46. doi: 10.1007/s40744-015-0014-y (PMC4883254; doi:10.1007/s40744-015-0014-y)
Supplement: Supplementary file 1 — Supplementary material 1 (PDF 189 kb) [file 40744_2015_14_MOESM1_ESM.pdf]

- Systemic lupus erythematosus has a complex pathogenesis and the course of events leading to this disease are not well understood.
- Exposure to certain environmental factors may impair the equilibrium between self and foreign as well as between tolerance and immune response, leading to autoimmune diseases, cancer and the so-called “lifestyle diseases”.
- These external stimuli may also alter the epigenetic status quo and may trigger lupus in genetically-susceptible individuals.
- Deciphering the contribution of epigenetic alterations to the pathogenesis of lupus will provide insights in this complex autoimmune disease.
- Epigenetic alterations are (potentially) reversible and hence promising candidates for the development of new therapeutics.

This summary slide represents the opinions of the authors. This work was supported by a grant of the ‘‘Rheinland-Pfalz Stiftung fur Innovation’’ (Grant 1032). For a full list of acknowledgments and conflicts of interest for all authors of this article, please see the full text online. Copyright © The Author(s) 2015. Creative Commons Attribution Noncommercial License (CC BY-NC).
